# Dengue Virus Serotype 3 Infection in Traveler Returning from West Africa to Germany

**DOI:** 10.3201/eid2101.141145

**Published:** 2015-01

**Authors:** Isabella Eckerle, Annette Kapaun, Thomas Junghanss, Paul Schnitzler, Christian Drosten, Thomas Jänisch

**Affiliations:** University of Bonn Medical Centre, Bonn, Germany (I. Eckerle, C. Drosten);; Heidelberg University Hospital, Heidelberg, Germany (A. Kapaun, T. Junghanss, P. Schnitzler, T. Jänisch)

**Keywords:** dengue virus, viruses, serotype 3, DENV-3, dengue, imported viral disease, traveler, phylogeny, West Africa, Germany

**To the Editor:** Dengue virus (DENV) is a member of the family *Flaviviridae*, genus *Flavivirus*, and comprises 4 serotypes (DENV-1, DENV-2, DENV-3, and DENV-4). DENV is transmitted by *Aedes* spp. mosquitoes in subtropical and tropical countries; an estimated 390 million dengue infections occurred worldwide in 2010 (*1*). In Africa, locally acquired dengue cases have been reported from 22 countries during 1960–2010, and there is evidence of transmission in 34 countries (*2*). The burden of dengue in Africa was recently estimated to be in the same range as that in Latin America (*1*).

In 2013, a 71-year-old man came to the Tropical Medicine Clinic at Heidelberg University Hospital (Heidelberg, Germany) with suspected dengue fever 3 days after returning from West Africa to Germany. The patient had traveled for ≈6 weeks from mid-September through mid-October 2013 to Togo (Lomié, first 3 weeks), Benin (Ouidah, 1 week), back to Togo (Lomié, 1 week), and Burkina Faso (Ouagadougou, 3 days).

Tests results for DENV nonstructural protein 1 and IgM against DENV were positive. The result of a real-time reverse transcription PCR for DENV 1–4 was also positive. A serotype-specific real-time reverse transcription PCR identified DENV-3 (*3*,*4*).

To obtain the sequence of a 1,479-nt fragment of the complete gene of the envelope glycoprotein gene, we designed generic primers specific for all complete DENV-3 genomes available from GenBank (alignment was performed by using Geneious version 6.1; http://www.geneious.com), which were then sequentially adapted to sequences obtained (primers and protocol available upon request). Sequencing of the complete envelope glycoprotein gene of the virus isolated from the patient identified DENV-3 genotype III (GenBank accession no. KJ922394).

For phylogenetic comparison, we chose all DENV-3 sequences available from Africa and neighboring regions and a set of global sequences that represented different genotypes; DENV-1 was used an outgroup. Sequences were aligned on the basis of translated nucleotide sequences, and a neighbor-joining tree with p-distance was inferred with 1,000 bootstrap replicates in MEGA version 5.2.1 (http://www.megasoftware.net/) for a 1,479-nt fragment spanning the complete gene of the envelope glycoprotein and a smaller fragment of 220 nt (Figure). We observed clustering of the virus sequence with those of strains from Côte d’Ivoire and Benin. Genetic identity was 99% with strains from Côte d’Ivoire (AB447989) (1,472/1,479 nt) and Benin (AB690858) (1,469/1,479 nt).

**Figure:**
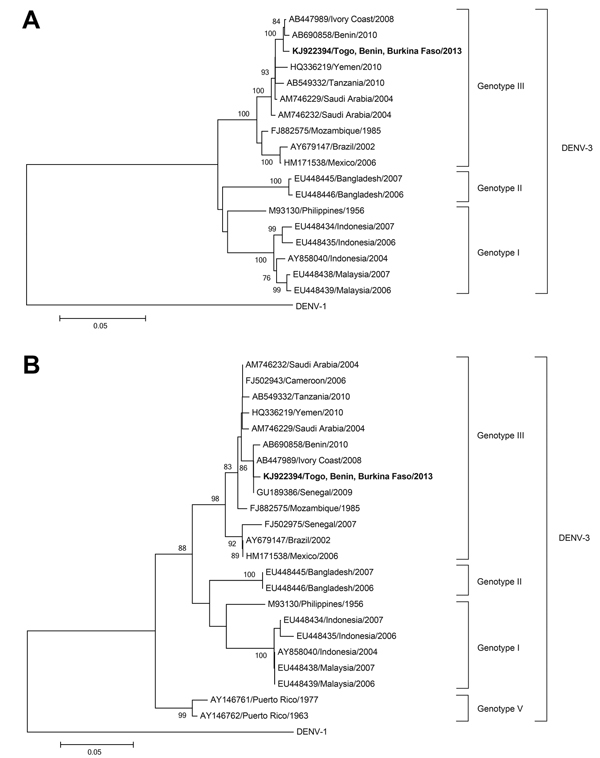
Phylogenetic trees of A) a 1,479-nt fragment of the complete envelope glycoprotein gene and B) a 220-nt fragment of the partial envelope glycoprotein gene of dengue virus. Phylogeny was based on a neighbor-joining tree with p-distance and 1,000 bootstrap replicates. Bootstrap values >75 are indicated along the branches. The strain isolated in this study from a 71-year-old man who returned from West Africa to Germany is indicated in bold. Scale bars indicate percentage of nucleotide distance.

Because of limited availability of only 4 complete envelope glycoprotein gene sequences from Africa, an additional phylogenetic analysis was performed with a smaller fragment of 220 nt to include more sequences of African origin. A total of 7 sequences, including additional sequences from Senegal and Cameroon, were available. Clustering of sequences of African origin was confirmed in this analysis; highest sequence identity of virus isolated from the patient was with viruses isolated in Côte d’Ivoire, Benin, and Senegal.

Nearly all sequence data for DENV-3 from Africa originate from returning travelers, such as reports of imported cases in 2006 from Cameroon to Spain (*5*), from Senegal to Spain in 2007 and to Italy in 2010 (*5*,*6*), from Côte d’Ivoire to France and Japan in 2008 (*7*,*8*), from Benin to Japan and France in 2010 (*8*,*9*), and from Eritrea to Finland in 2010 (*5*). Phylogenetic analysis of virus strains identified in these cases shows clustering with sequences of African origin, as observed for our patient (*6*–*8*). However, because only a small number of sequences of DENV-3 strains from Africa are available (and not all sequences refer to the same genomic region), a comparative phylogenetic analysis of strains from Africa is limited. A recent investigation of febrile patients from Gabon showed not only circulation of DENV-3, but simultaneous circulation of 3 DENV serotypes (DENV-1, DENV-2, and DENV-3) in West Africa (*10*).

Molecular data for travelers are useful in areas where DENV diagnosis and surveillance are not routinely performed. The case-patient reported here highlights sustained transmission of DENV-3 genotype III strains or closely related strains during recent years. Increasing numbers of reports on local outbreaks and available phylogenetic information support ongoing DENV-3 transmission in West Africa. If one assumes a maximum incubation time of 14 days, our case-patient was most probably exposed to DENV in Togo or Burkina Faso. These 2 countries have not been considered as areas to which DENV is endemic. Our findings indicate that further systematic evaluation of the risk and disease burden of dengue in Africa is urgently needed. Dengue fever should be considered in travelers returning from Africa with acute febrile illness.
